# Association of postoperative psychological interventions on compliance and survival in breast cancer patients: A single-center retrospective study

**DOI:** 10.1097/MD.0000000000048166

**Published:** 2026-04-10

**Authors:** Rui Dong, Jingjing Yu

**Affiliations:** a Endocrine Surgery, Xingtai Central Hospital, Xingtai City, Hebei Province, China; b Endocrine Surgery/Medical Aesthetic Surgery, Xingtai Central Hospital, Xingtai City, Hebei Province, China.

**Keywords:** adjuvant endocrine therapy, breast cancer, disease-free survival, medication adherence, overall survival, postoperative psychological intervention, proportion of days covered, psycho-oncology

## Abstract

This study aimed to whether different patterns of postoperative psychological intervention were associated with subsequent adherence to adjuvant endocrine therapy and survival in women with hormone receptor-positive (HR+) breast cancer. We conducted a single-center retrospective cohort study including 318 women with stage I–III, HR+ breast cancer who underwent curative-intent surgery between June 2022 and June 2024 at a tertiary hospital in China. Psycho-oncology care delivered within 6 months of surgery was grouped into 3 patterns: no/minimal care (0–1 session), early structured care (≥3 sessions starting within 3 months) and delayed/low-intensity care (≥3 sessions starting > 3–6 months, or 2 sessions spread over >3 months). Overall survival and disease-free survival were evaluated using a 6-month landmark approach. Associations between intervention patterns and outcomes were assessed with logistic regression and Cox models incorporating inverse probability of treatment weighting based on multinomial propensity scores. In total, 215 of 318 patients (67.6%) met the definition of good 1-year adherence. The proportion with good adherence was higher in the early structured (77.0%) and delayed/low-intensity groups (71.6%) than in the no/minimal group (55.7%). Relative to no/minimal care, early structured intervention was associated with a significantly greater odds of good adherence (adjusted odds ratio [OR] 2.15, 95% confidence interval [CI] 1.27–3.64; *P* = .004), and delayed/low-intensity intervention showed a more modest but still favorable association (adjusted OR 1.81, 95% CI 1.01–3.24; *P* = .045). In inverse probability of treatment weighting analyses, the ORs for good adherence were 2.05 (95% CI 1.23–3.42; *P* = .006) for early structured and 1.67 (95% CI 0.95–2.94; *P* = .075) for delayed/low-intensity care compared with no/minimal care. After a median follow-up of 24.1 months, early structured intervention was associated with lower,. In this postoperative cohort of women with stage I–III, HR+ breast cancer, early structured psychological intervention was independently linked with better 1-year adherence to adjuvant endocrine therapy. Apparent advantages for overall survival and disease-free survival were consistent in direction but imprecise. The findings support embedding structured psycho-oncology care into routine postoperative follow-up to address distress and help patients maintain long-term endocrine therapy.

## 1. Introduction

Breast cancer is the most commonly diagnosed malignancy among women and remains a leading cause of cancer-related death worldwide.^[1]^ Improvements in screening, surgical techniques and systemic therapies have led to substantial gains in long-term survival, particularly in high-income countries.^[2]^ For women with stage I–III disease, breast-conserving surgery or mastectomy combined with risk-adapted adjuvant chemotherapy, radiotherapy, endocrine therapy and human epidermal growth factor receptor 2 (HER2)-targeted therapy is now standard of care.^[2]^ In hormone receptor-positive (HR+) breast cancer, prolonged adjuvant endocrine therapy (AET) for 5 to 10 years can significantly reduce recurrence and mortality, although the absolute benefit depends on baseline risk and treatment duration.^[3]^ However, the magnitude of this benefit in practice depends critically on patients’ adherence to complex, long-term treatment plans.^[4]^

Diagnosis and surgery for breast cancer are profound psychological stressors. Many patients experience marked anxiety, depressive symptoms, fear of recurrence and body-image concerns in the postoperative period, and a substantial proportion continue to report psychological distress for years after primary treatment.^[5]^ Psychological morbidity is not only a quality-of-life issue. Large cohort and pooled analyses suggest that depression and anxiety are associated with higher risks of recurrence and all-cause mortality, even after adjustment for established clinical prognostic factors.^[6]^ In addition, patients with significant psychological burden or preexisting mental illness are less likely to receive or complete guideline-concordant oncological treatment, including adjuvant systemic therapies.^[7]^

In response to this burden, a wide range of psychosocial and psychological interventions have been developed for women with breast cancer, including cognitive-behavioral therapy, supportive-expressive group therapy, mindfulness-based programs and structured psychoeducation.^[8]^ Systematic reviews and meta-analyses consistently demonstrate that such interventions reduce symptoms of anxiety and depression and improve health-related quality of life across the disease trajectory.^[8,9]^ More recent work has shown that psychosocial interventions can attenuate biological stress responses, for example by reducing cortisol levels, suggesting plausible biopsychosocial mechanisms linking psychological care with oncological outcomes.^[10]^ However, evidence regarding their association on “hard” oncological endpoints is less consistent. A meta-analysis of randomized trials reported short-term improvements in survival after psychosocial interventions, particularly group-based programs, but no clear benefit at longer follow-up. Other meta-analyses failed to demonstrate a robust survival advantage, and survival benefits, when observed, tend to be time-limited and not consistently replicated across trials.^[10,11]^ Consequently, the extent to which postoperative psychological interventions influence long-term survival remains uncertain.

One key pathway through which psychological interventions may affect outcome is their association on treatment adherence. Long-term adherence to AET is crucial for patients with HR+ breast cancer, yet real-world adherence is frequently suboptimal.^[4,5,11]^ Recent systematic review data indicate that a substantial proportion of women are non-adherent or discontinue AET prematurely, and that these patients face significantly higher risks of recurrence and mortality compared with adherent patients.^[5]^ Psychosocial factors such as depressive symptoms, maladaptive coping, negative beliefs about medication and low self-efficacy have been repeatedly linked to poor adherence. Interventions targeting illness perceptions, expectations about endocrine therapy, coping with side effects and patient empowerment – including psychoeducational and coping-skills programs – have shown promise in improving adherence in randomized and quasi-experimental studies.^[11]^ Nonetheless, most studies have been conducted in selected survivorship samples or experimental settings, and little is known about the effect of postoperative psychological care, as routinely delivered in clinical practice, on objective measures of AET adherence and long-term survival.

Taken together, current evidence suggests that postoperative psychological distress is common after breast cancer surgery, is associated with reduced adherence to adjuvant therapy and may be linked to poorer survival, whereas structured psychological interventions reliably improve emotional and quality-of-life outcomes but have uncertain effects on survival.^[12]^ There is a clear need for data examining whether psychological interventions received in the postoperative period are associated with better adherence to long-term endocrine therapy and improved oncological outcomes in contemporary surgical cohorts. Therefore, the present study aimed to investigate, in a single-center cohort of women undergoing surgery for stage I–III breast cancer, the association of postoperative psychological interventions on adherence to AET and overall and disease-free survival (DFS). By integrating epidemiological, surgical, psychosocial and behavioral perspectives, this study seeks to provide clinically relevant evidence on the potential prognostic importance of embedding structured psychological care into routine postoperative management of breast cancer patients.

## 2. Materials and methods

### 2.1. Study design and setting

This study was approved by the Ethics Committee of Xingtai Central Hospital (No. 2025-KY-07). This single-center retrospective cohort study was conducted at the Department of Breast Surgery of a tertiary hospital in China. The department provides guideline-based multidisciplinary care, including surgery, systemic therapy, radiotherapy and a routine distress-screening and psycho-oncology program integrated into postoperative follow-up.

### 2.2. Study population

We identified all consecutive women who underwent curative-intent surgery for primary breast cancer between June 1, 2022 and June 30, 2024. A total of 318 patients fulfilled these criteria and were included in the analysis. We restricted the cohort to patients with HR+ invasive breast cancer because long-term adherence to AET is the main focus of this study.

### 2.3. Inclusion criteria

Eligible patients met all of the following criteria:

1. Histologically confirmed invasive breast carcinoma;
2. Stage I–III disease at diagnosis according to the American Joint Committee on Cancer 7th edition;
3. HR+ (ER and/or PR+) invasive breast carcinoma;
4. Breast-conserving surgery or mastectomy with curative intent, with or without immediate reconstruction;
5. Age 18 to 80 years at the time of surgery;
6. At least one documented postoperative psychological distress assessment within 6 weeks after surgery;
7. Availability of electronic medical records, pharmacy refill data and follow-up information up to June 30, 2025 or death, whichever occurred first.

### 2.4. Exclusion criteria

Patients were excluded if they had:

1. De novo stage IV or recurrent breast cancer at presentation;
2. Ductal carcinoma in situ only;
3. A previous invasive malignancy within 5 years (excluding adequately treated non-melanoma skin cancer or cervical carcinoma in situ);
4. preexisting severe psychotic disorder requiring long-term institutional care;
5. Death, locoregional recurrence or distant metastasis within 3 months after surgery;
6. Refusal of all adjuvant oncologic treatment or loss to follow-up within 12 months after surgery.

### 2.5. Follow-up

Patients were followed from the date of curative surgery until death or the data cutoff of June 30, 2025, whichever occurred first. Because accrual was limited to June 1, 2022–June 30, 2024, all patients had at least 12 months of postoperative follow-up, and the earliest cases had up to ~3 years of follow-up. Adherence to AET was evaluated during the first year after initiation; survival outcomes were assessed until the data cutoff.

### 2.6. Postoperative psycho-oncology care

Postoperative psychological care was delivered by a dedicated psycho-oncology team comprising psychiatrists and clinical psychologists. Care was offered according to routine distress screening and clinical judgement and included:

① Individual supportive or cognitive–behavioral sessions,

② Group-based psychoeducational and support programs,

③ Brief mindfulness or relaxation training,

④ Family sessions when indicated.

Interventions followed semi-structured manuals but were tailored to individual needs. Sessions usually lasted 30 to 60 minutes and were scheduled every 2 to 4 weeks during active treatment, then as required. For each patient, the psycho-oncology database was reviewed to obtain the dates and number of sessions, format (individual vs group) and main modality (cognitive–behavioral, supportive, psychoeducational, mindfulness/relaxation) within the first 6 months after surgery.

### 2.7. Exposure: patterns of postoperative psychological

Patterns of postoperative psychological care in the first 6 months after surgery were classified a priori as: no/minimal intervention: 0 to 1 documented psycho-oncology session; early structured intervention: ≥3 sessions initiated within 3 months of surgery; and delayed/low-intensity intervention: ≥3 sessions initiated >3 and ≤6 months after surgery, or 2 sessions spread over >3 months within the first 6 months. The primary comparison was early structured intervention versus no/minimal intervention. The delayed/low-intensity category was examined separately to explore potential effects of timing and intensity.

### 2.8. Distress assessment and trajectories

Psychological distress was routinely assessed at 4 postoperative time points:

Within 6 weeks after surgery (baseline), ~3 months, 6 months, 12 months. Three-class solutions were selected for both distress and adherence trajectories based on the lowest Bayesian information criterion, adequate average posterior probabilities (>0.7) and clinical interpretability. At each time point, patients completed the Hospital Anxiety and Depression Scale (HADS) and the National Comprehensive Cancer Network distress thermometer. Scores were retrieved from the electronic medical record. To describe distinct patterns of distress over time, latent class mixed models were fitted to repeated HADS total scores to identify distress trajectory classes (e.g., low-stable, improving, high-persistent). The number of classes was determined using Bayesian information criteria, posterior probabilities and clinical interpretability. Distress trajectories were used descriptively and as potential effect modifiers in subsequent analyses.

### 2.9. Adjuvant treatment and endocrine therapy

Adjuvant chemotherapy, endocrine therapy, HER2-targeted therapy and radiotherapy were administered according to contemporary national guidelines, based on tumor characteristics, stage and comorbidities. For HR+ disease, AET with tamoxifen and/or aromatase inhibitors was recommended for at least 5 years, with possible extension in higher-risk patients. The AET start date was defined as the date of the first prescription after completion of adjuvant chemotherapy, or within 3 months of surgery in patients who did not receive chemotherapy. Switching between tamoxifen and aromatase inhibitors was allowed; endocrine therapy was considered continuous if gaps between prescriptions were ≤ 90 days.

### 2.10. Adherence measurement and trajectories

Adherence to AET was assessed from pharmacy dispensing records, with the first 12 months after AET initiation defined as the primary observation period.

1. Monthly proportion of days covered (PDC): For each 30-day interval during the first year, PDC was calculated as the number of days with AET available divided by 30.
2. Adherence trajectories: Group-based trajectory modeling was applied to monthly PDC values to derive adherence patterns over the first year (such as high-stable, moderate-declining, early-discontinuation). The number of trajectories was chosen according to model fit indices and clinical plausibility.
3. Binary adherence endpoint: For the primary endpoint, good adherence was defined as PDC ≥ 80% in at least 80% of observed months during the first 12 months of AET and no medication gap >90 days. All other patients were classified as having poor adherence.

Binary adherence endpoint: For the primary endpoint, good adherence was defined as a PDC ≥ 80% in at least 80% of observed months during the first 12 months of AET and no medication gap >90 days. This composite definition was chosen for several reasons. First, a PDC threshold of ≥80% is a widely accepted standard in pharmacoepidemiology, often used to distinguish adherent from non-adherent patients as it correlates with clinical effectiveness. Second, requiring this level of adherence in at least 80% of the months captures not only the average adherence over the year but also the consistency of medication-taking behavior, thereby identifying patients who maintained good adherence throughout most of the follow-up period. Finally, the inclusion of a “no gap >90 days” criterion helps to flag patients with premature discontinuation, a clinically critical event. This multifaceted definition provides a more robust and clinically meaningful measure of good adherence than a simple average PDC over 12 months. All other patients were classified as having poor adherence.

### 2.11. Survival outcomes

Two oncologic outcomes were evaluated: overall survival (OS): time from surgery to death from any cause or last contact; DFS: time from surgery to first ipsilateral or contralateral breast recurrence, regional nodal recurrence, distant metastasis, second primary invasive cancer or death.

Because patterns of psycho-oncology care during the first 6 months after surgery are time-dependent, and to minimize immortal time bias, we used a 6-month landmark design for survival analyses, including only patients alive and recurrence-free at 6 months and defining exposure according to psychological care received within the first 6 months. Recurrence events were identified from clinical notes, imaging and pathology reports. Vital status and dates of death were obtained from hospital records and regional registries when available.

### 2.12. Covariates

Baseline variables extracted from the medical record included:

1. Demographic: age, menopausal status, marital status, employment status;
2. Clinical: body mass index, Charlson comorbidity index, family history of breast cancer;
3. Tumor-related: histological type, grade, tumor size (T stage), nodal status (N stage), overall stage (I vs II–III), HR status, HER2 status, Ki-67 index;
4. Treatment-related: type of breast surgery (breast-conserving vs mastectomy), axillary procedure, receipt of chemotherapy, radiotherapy and HER2-targeted therapy, and initial AET agent;
5. Psychological: baseline HADS anxiety and depression scores, recorded psychiatric history, baseline use of antidepressants or anxiolytics;
6. Calendar time: year of surgery.

### 2.13. Data sources and quality control

Three institutional databases were linked using unique patient identifiers: the electronic medical record (demographic, clinical, tumor and treatment data), the psycho-oncology database (session-level information) and the hospital pharmacy database (all AET dispensings). Data extraction followed a predefined data dictionary. Two trained research assistants independently abstracted data; a random 10% sample was reviewed by a senior investigator. Logical checks (e.g., chronological order of diagnosis, surgery and adjuvant therapies; absence of endocrine prescriptions before diagnosis) were performed, and discrepancies were resolved by reference to the original records.

### 2.14. Statistical analysis

All analyses were performed using R version 4.3.2 (R Foundation for Statistical Computing, Vienna, Austria). Continuous variables are presented as means with standard deviations or medians with interquartile ranges, and categorical variables as counts and percentages. Between-group comparisons used χ^2^ or Fisher’s exact tests for categorical variables and t tests or non-parametric tests for continuous variables, as appropriate. Latent class mixed models (for repeated HADS total scores) and group-based trajectory modeling (for monthly PDC values) were used to derive psychological distress and adherence trajectories. Three-class solutions were selected for both distress and adherence trajectories based on Bayesian information criterion, average posterior probabilities (>0.7) and clinical interpretability. The association between patterns of postoperative psychological intervention and good 1-year AET adherence was examined using logistic regression, with additional models using inverse probability of treatment weighting (IPTW) based on propensity scores to reduce confounding. Propensity scores for the 3 intervention patterns were estimated from multinomial logistic regression including key demographic, clinical, tumor and treatment covariates; stabilized IPTWs were then applied in weighted logistic and Cox models. OS and DFS from a 6-month landmark (including patients alive and recurrence-free at 6 months, with exposure defined by psychological care received within the first 6 months) were described by Kaplan–Meier curves and compared using Cox proportional hazards models, with IPTW applied in primary adjusted analyses. Exploratory mediation and subgroup analyses were conducted to investigate the potential roles of adherence and distress trajectories. Missing covariate data were handled by complete-case analysis, with sensitivity analyses using multiple imputation by chained equations. All statistical tests were 2-sided, and *P* < .05 was considered statistically significant.

### 2.15. Sample size and power

The primary endpoint was good 1-year adherence to AET among HR+ patients. On the basis of a 2-sample comparison of proportions, assuming a 1-year adherence rate of 60% in the usual-care group and 75% in the early structured intervention group (absolute difference 15% points), with a 2-sided α of 0.05 and 80% power, a total sample of ~300 to 320 patients would be required. The final cohort of 318 patients falls within this range and was therefore considered adequate for the primary adherence analysis. Given the limited follow-up and the expected number of recurrences and deaths, analyses of OS and DFS were regarded as descriptive and exploratory.

### 2.16. Ethical approval

The study protocol was approved by the institutional ethics committee. Owing to the retrospective design and use of de-identified data, the requirement for written informed consent was waived. The study was conducted in accordance with the Declaration of Helsinki and relevant national regulations on data protection.

## 3. Results

### 3.1. Patient characteristics

During the study period, 318 women fulfilled the eligibility criteria and were included in the analysis. The median age at diagnosis was 52 years (interquartile range [IQR] 45–60); 42.5% were younger than 50 years, 34.6% were 50 to 59 years and 23.0% were ≥ 60 years. Most patients had stage I (42.1%) or II (39.9%) disease, and 17.9% had stage III disease; 66.0% underwent breast-conserving surgery and 34.0% mastectomy. All tumors were HR+, and 22.6% were HER2-positive. Adjuvant chemotherapy and radiotherapy were administered to 70.4% and 80.5% of patients, respectively, and all initiated AET with tamoxifen and/or an aromatase inhibitor. Baseline psychological distress was modestly elevated (median HADS total 13, IQR 9–18). Baseline demographic, tumor and treatment characteristics were broadly comparable across psychological intervention patterns, although patients receiving early structured intervention were slightly younger and more likely to have received chemotherapy (Table 1).

**Table 1:** Baseline demographic, clinical and tumor characteristics according to postoperative psychological intervention pattern.

Characteristic	Total (n = 318)	No/minimal (n = 122)	Early structured (n = 122)	Delayed/low-intensity (n = 74)	*P* value*
Age (yr), mean ± SD	52.1 ± 9.8	53.0 ± 10.1	50.8 ± 9.4	52.5 ± 9.6	.18
Age <50 yr, n (%)	135 (42.5)	45 (36.9)	61 (50.0)	29 (39.2)	.09
Postmenopausal, n (%)	176 (55.3)	73 (59.8)	61 (50.0)	42 (56.8)	.29
Married/partnered, n (%)	262 (82.4)	101 (82.8)	98 (80.3)	63 (85.1)	.77
Employed, n (%)	179 (56.3)	64 (52.5)	75 (61.5)	40 (54.1)	.36
Charlson comorbidity index ≥1, n (%)	88 (27.7)	38 (31.1)	28 (23.0)	22 (29.7)	.30
BMI, kg/m^2^, mean ± SD	24.7 ± 3.6	24.9 ± 3.7	24.4 ± 3.5	24.8 ± 3.7	.53
Family history of breast cancer, n (%)	64 (20.1)	25 (20.5)	24 (19.7)	15 (20.3)	.99
Stage I, n (%)	134 (42.1)	50 (41.0)	55 (45.1)	29 (39.2)	.81
Stage II, n (%)	127 (39.9)	50 (41.0)	46 (37.7)	31 (41.9)	
Stage III, n (%)	57 (17.9)	22 (18.0)	21 (17.2)	14 (18.9)	
Tumor grade 3, n (%)	94 (29.6)	37 (30.3)	36 (29.5)	21 (28.4)	.96
Node-positive disease, n (%)	150 (47.2)	55 (45.1)	60 (49.2)	35 (47.3)	.84
HER2-positive, n (%)	72 (22.6)	28 (23.0)	27 (22.1)	17 (23.0)	.98
Ki-67 ≥ 20%, n (%)	182 (57.2)	67 (54.9)	75 (61.5)	40 (54.1)	.47
Breast-conserving surgery, n (%)	210 (66.0)	77 (63.1)	84 (68.9)	49 (66.2)	.63
Chemotherapy, n (%)	224 (70.4)	78 (63.9)	92 (75.4)	54 (73.0)	.09
Radiotherapy, n (%)	256 (80.5)	95 (77.9)	104 (85.2)	57 (77.0)	.23
HER2-targeted therapy, n (%)†	59 (18.6)	23 (18.9)	21 (17.2)	15 (20.3)	.85
Initial AET: aromatase inhibitor, n (%)	178 (56.0)	67 (54.9)	71 (58.2)	40 (54.1)	.80
HADS total at baseline, median (IQR)	13 (9–18)	12 (8–17)	14 (10–18)	13 (9–18)	.11

AET = adjuvant endocrine therapy, BMI = body mass index, HADS = Hospital Anxiety and Depression Scale, HER2 = human epidermal growth factor receptor 2, IQR = interquartile range, SD = standard deviation.

* *P* values from χ^2^, Fisher’s exact, ANOVA or Kruskal–Wallis tests as appropriate.

† Among HER2-positive patients only.

### 3.2. Uptake and patterns of postoperative psychological intervention

Of the 318 patients, 122 (38.4%) received early structured postoperative psychological intervention, 74 (23.3%) received delayed/low-intensity intervention, and 122 (38.4%) had no or minimal intervention within the first 6 months after surgery. In the early structured group, the median number of sessions was 5 (IQR 4–7), and 66.4% attended at least 5 sessions; 68.0% received cognitive–behavioral–oriented individual sessions, 74.6% supportive sessions and 54.9% psychoeducational or support groups. In the delayed/low-intensity group, the median number of sessions was 3 (IQR 2–4), predominantly supportive or psychoeducational rather than formally cognitive–behavioral. Overall, 192 patients (60.4%) received at least one psycho-oncology session, most commonly prompted by elevated distress at baseline or at the 3-month assessment (Table 2).

**Table 2:** Uptake and characteristics of postoperative psychological intervention.

Variable	No/minimal (n = 122)	Early structured (n = 122)	Delayed/low-intensity (n = 74)	*P* value
≥1 psycho-oncology session, n (%)	0 (0.0)	122 (100)	70 (94.6)	<.001
Sessions within 6 mo, median (IQR)	0 (0–1)	5 (4–7)	3 (2–4)	<.001
Sessions ≥ 5, n (%)	0 (0.0)	81 (66.4)	21 (28.4)	<.001
First session ≤1 mo after surgery, n (%)	0 (0.0)	74 (60.7)	9 (12.2)	<.001
First session 1–3 mo after surgery, n (%)	0 (0.0)	48 (39.3)	22 (29.7)	
First session >3–6 mo after surgery, n (%)	0 (0.0)	0 (0.0)	39 (52.7)	
Any CBT-oriented individual session, n (%)	0 (0.0)	83 (68.0)	32 (43.2)	<.001
Any supportive individual session, n (%)	0 (0.0)	91 (74.6)	48 (64.9)	.17
Any psychoeducational/support group, n (%)	0 (0.0)	67 (54.9)	29 (39.2)	.03
Any mindfulness/relaxation training, n (%)	0 (0.0)	46 (37.7)	21 (28.4)	.20
Family sessions, n (%)	0 (0.0)	18 (14.8)	8 (10.8)	.42

*P* values from χ^2^ or Kruskal–Wallis tests as appropriate.

CBT = cognitive–behavioral therapy, IQR = interquartile range.

### 3.3. Psychological distress trajectories

Latent class mixed modeling of repeated HADS total scores identified 3 psychological distress trajectories over 12 months. The low-stable class (n = 160, 50.3%) had low baseline distress (median HADS 9) that remained low. The improving class (n = 90, 28.3%) showed moderately elevated baseline distress (median HADS 16) with marked symptom reduction by 6–12 months. The high-persistent class (n = 68, 21.4%) had high baseline distress (median HADS 21) and persistently elevated scores at 12 months (median 18). Early structured psychological intervention was more frequent in the improving class, whereas no or minimal intervention predominated in the high-persistent class. In multinomial models, compared with no/minimal intervention, early structured intervention was associated with higher odds of belonging to the improving rather than the high-persistent trajectory (adjusted odds ratio [OR] 1.84, 95% confidence interval [CI] 1.02–3.32), while its association with the low-stable trajectory was weaker and not significant (Table 3).

**Table 3:** Psychological distress trajectories and baseline features.

Distress trajectory class	n (%)	Baseline HADS total, median (IQR)	12-mo HADS total, median (IQR)	Early structured intervention, n (%)	No/minimal intervention, n (%)
Low-stable	160 (50.3)	9 (7–11)	8 (6–10)	53 (33.1)	63 (39.4)
Improving	90 (28.3)	16 (14–19)	9 (7–11)	48 (53.3)	20 (22.2)
High-persistent	68 (21.4)	21 (19–24)	18 (16–21)	21 (30.9)	39 (57.4)

*P* < .001 for differences in baseline HADS, 12-month HADS and distribution of intervention patterns across trajectories.

Trajectories derived from latent class mixed models fitted to repeated HADS total scores.

HADS = Hospital Anxiety and Depression Scale, IQR = interquartile range.

### 3.4. Adherence trajectories and primary AET adherence outcome

During the first 12 months of AET, group-based trajectory modeling identified 3 adherence patterns. The high-stable class (n = 198, 62.3%) maintained PDC values ≥90% throughout the year. The moderate-declining class (n = 76, 23.9%) started with PDC around 80% to 90% and declined to 60% to 70% by month 12. The early-discontinuation class (n = 44, 13.8%) showed a rapid fall in PDC to <50% within the first 6 months, often with prolonged gaps. Overall, the mean 1-year PDC was 0.84 (standard deviation 0.15). Using the predefined binary endpoint, 215 patients (67.6%) met criteria for good adherence and 103 (32.4%) had poor adherence. Good adherence was more frequent in the early structured (77.0%, 94/122) and delayed/low-intensity groups (71.6%, 53/74) than in the no/minimal group (55.7%, 68/122), with corresponding mean PDC values of 0.88, 0.86, and 0.79, respectively (Table 4).

**Table 4:** One-year AET adherence and adherence trajectories by psychological intervention pattern.

Variable	Total (n = 318)	No/minimal (n = 122)	Early structured (n = 122)	Delayed/low-intensity (n = 74)	*P* value
Mean PDC in first year, mean ± SD	0.84 ± 0.15	0.79 ± 0.17	0.88 ± 0.13	0.86 ± 0.14	<.001
Good adherence*, n (%)	215 (67.6)	68 (55.7)	94 (77.0)	53 (71.6)	<.001
High-stable trajectory, n (%)	198 (62.3)	61 (50.0)	86 (70.5)	51 (68.9)	.002
Moderate-declining trajectory, n (%)	76 (23.9)	35 (28.7)	25 (20.5)	16 (21.6)	
Early-discontinuation trajectory, n (%)	44 (13.8)	26 (21.3)	11 (9.0)	7 (9.5)	

AET = adjuvant endocrine therapy, PDC = proportion of days covered, SD = standard deviation.

* Good adherence defined as PDC ≥80% in ≥80% of months and no gap >90 days during the first 12 months of AET.

### 3.5. Association between psychological intervention and AET adherence

In unadjusted analyses, early structured intervention was associated with higher odds of good 1-year AET adherence compared with no/minimal intervention (OR 2.67, 95% CI: 1.57–4.53; *P* < .001), and delayed/low-intensity intervention was also favorable (OR 1.94, 95% CI: 1.08–3.47; *P* = .026). After adjustment for age, stage, nodal status, comorbidity, systemic therapy, HER2 status, initial endocrine agent and baseline HADS score, early structured intervention remained independently associated with good adherence (adjusted OR 2.15, 95% CI: 1.27–3.64; *P* = .004), and delayed/low-intensity intervention showed a weaker but still significant association (adjusted OR 1.81, 95% CI: 1.01–3.24; *P* = .045). In IPTW models based on multinomial propensity scores, early structured intervention was associated with a 2.05-fold higher odds of good adherence versus no/minimal intervention (95% CI: 1.23–3.42; *P* = .006), whereas the corresponding estimate for delayed/low-intensity intervention was 1.67 (95% CI: 0.95–2.94; *P* = .075; Table 5).

**Table 5:** Logistic regression analysis of psychological intervention patterns and good 1-year adherence.

Exposure comparison	Crude OR (95% CI)	*P* value	Adjusted OR* (95% CI)	*P* value	IPTW-weighted OR† (95% CI)	*P* value
Early structured vs no/minimal	2.67 (1.57–4.53)	<.001	2.15 (1.27–3.64)	.004	2.05 (1.23–3.42)	.006
Delayed/low-intensity vs no/minimal	1.94 (1.08–3.47)	.026	1.81 (1.01–3.24)	.045	1.67 (0.95–2.94)	.075
Any psycho-oncology care vs no/minimal	2.20 (1.37–3.54)	.001	1.98 (1.20–3.26)	.007	1.90 (1.16–3.10)	.011

AET = adjuvant endocrine therapy, CI = confidence interval, HADS = Hospital Anxiety and Depression Scale, HER2 = human epidermal growth factor receptor 2, IPTW = inverse probability of treatment weighting, OR = odds ratio.

* Adjusted for age, stage, nodal status, Charlson comorbidity index, chemotherapy, radiotherapy, HER2 status, initial endocrine agent and baseline HADS total score.

† Inverse probability of treatment weighting based on multinomial propensity scores.

### 3.6. Survival outcomes

At data cutoff, the median follow-up from surgery was 24.1 months (IQR 18.3–30.4). Overall, 34 DFS events (10.7%) and 22 deaths (6.9%) had occurred. In the 6-month landmark cohort (patients alive and recurrence-free at 6 months), the estimated 2-year OS rates were 96.0% in the early structured group, 93.5% in the delayed/low-intensity group and 91.0% in the no/minimal group; the corresponding 2-year DFS rates were 92.4%, 89.0% and 85.2%, respectively. In IPTW-weighted Cox models, early structured intervention was associated with a lower, but not statistically significant, hazard of death compared with no/minimal intervention (hazard ratio [HR] for OS 0.58, 95% CI: 0.22–1.54; *P* = .27), with a similar pattern for DFS (HR 0.61, 95% CI: 0.30–1.23; *P* = .17). Delayed/low-intensity intervention yielded intermediate HRs. Further adjustment for AET adherence attenuated these associations, suggesting that any potential survival benefit of early structured intervention may be partly mediated through improved endocrine therapy adherence (Table 6).

**Table 6:** Overall and disease-free survival according to psychological intervention pattern (6-month landmark analysis).

Outcome	No/minimal (n = 118)*	Early structured (n = 118)*	Delayed/low-intensity (n = 72)*	*P* value†
Median follow-up (mo)	23.5	24.6	24.0	.48
OS events, n (%)	10 (8.5)	5 (4.2)	7 (9.7)	.23
DFS events, n (%)	14 (11.9)	9 (7.6)	11 (15.3)	.19
2-yr OS, % (95% CI)	91.0 (85.1–97.0)	96.0 (92.0–100)	93.5 (87.1–99.8)	.21‡
2-yr DFS, % (95% CI)	85.2 (77.8–92.6)	92.4 (87.4–97.4)	89.0 (81.1–96.9)	.18‡
IPTW HR for OS vs no/minimal	–	0.58 (0.22–1.54)	0.91 (0.39–2.09)	
IPTW HR for DFS vs no/minimal	–	0.61 (0.30–1.23)	0.94 (0.48–1.83)	

CI = confidence interval, DFS = disease-free survival, HR = hazard ratio, IPTW = inverse probability of treatment weighting, OS = overall survival.

* Numbers at risk at the 6-month landmark (patients alive and recurrence-free).

† *P* values for differences in event proportions from χ^2^ tests.

‡ *P* values from IPTW-weighted log-rank tests.

### 3.7. Sensitivity analyses

Findings were generally robust in sensitivity analyses. Excluding 12 patients who developed a new major psychiatric disorder after surgery did not materially change the association between psychological intervention patterns and adherence. Using a stricter definition of good adherence (PDC ≥ 90% in ≥ 80% of months) reduced the overall adherence rate to 54.4%, but the relative effect of early structured intervention was similar (adjusted OR 2.07, 95% CI 1.21–3.54).

When psychological intervention was modeled as a time-varying exposure in extended Cox models, the estimated effects on OS and DFS were directionally consistent with the landmark analysis, although precision was limited by the small number of events. Complete-case analyses and multiple imputation for missing covariates (<7% for any variable) produced very similar results.

## 4. Discussion

In this single-center retrospective cohort, women who received early, structured postoperative psychological care were more likely to adhere to AET during the first year after treatment than those receiving no or minimal psycho-oncology input. The association remained after adjustment for a wide range of clinical and treatment factors and was broadly consistent in IPTW analyses. By contrast, any survival advantage appeared modest and did not reach statistical significance, which is not unexpected given the limited follow-up and small number of outcome events. Together, these findings suggest that postoperative psychological care, when delivered in a structured and timely manner, may play a meaningful role in supporting long-term endocrine therapy use, while its effect on survival requires further study.

It is important to clarify that the classification of psychological intervention in this study – “early structured,” “delayed/low-intensity,” and “no/minimal” – reflects distinct patterns of psycho-oncology service delivery as they occurred in our real-world clinical practice. These patterns were defined based on the timing and quantity of sessions, not by a prespecified, manualized intervention protocol. Therefore, the observed associations should be interpreted as reflecting the potential benefit of engaging with a structured, routine psycho-oncology service in the postoperative period, rather than the efficacy of a specific psychological technique.

The distress patterns observed in this cohort are in line with previous work showing that a substantial proportion of women experience clinically relevant anxiety and depressive symptoms around the time of breast cancer diagnosis and surgery, and that symptoms often persist beyond completion of primary treatment.^[6,7]^ The 3 trajectories identified – low-stable, improving and high-persistent distress – resemble those described in earlier longitudinal studies, which also found a smaller subgroup with chronically high symptom burden.^[12]^ In our data, early structured psychological intervention was more frequent in women whose distress improved over time, whereas no or minimal care was common among those with sustained high distress. This pattern is clinically important: the patients most likely to benefit from intensive psychological support may be the ones who are least likely to receive or engage with it.

The positive association between early structured intervention and improving distress is consistent with evidence from randomized trials and meta-analyses showing that various psychosocial interventions can reduce distress and improve quality of life in women with early-stage breast cancer.^[13]^ At the same time, the apparent under-representation of the high-persistent group among recipients of early structured care highlights potential gaps in identification, referral and uptake. Routine distress screening, coupled with proactive follow-up of high scores and flexible delivery formats (for example, brief sessions, telephone or online contacts), may help close this gap in busy postoperative clinics.^[10]^

Approximately one-third of women in our study did not achieve good adherence to AET over the first year. This is comparable to estimates from other observational cohorts, which report substantial rates of non-adherence and early discontinuation and link these behaviors to poorer disease outcomes.^[14,15]^ Our adherence trajectories – high-stable, moderate-declining and early-discontinuation – provide a pragmatic framework for recognizing patients whose adherence is beginning to erode or deteriorates rapidly, and who may need additional support early in the course of treatment.^[16]^ Within this framework, early structured psychological care was associated with a more favorable adherence profile, whereas patients with no or minimal psycho-oncology input were more likely to follow a moderate-declining or early-discontinuation trajectory.

Interventions to improve adherence to endocrine therapy in breast cancer are diverse and often multifaceted, combining education, reminders, structured follow-up and counseling.^[11,17]^ Many of these programs, however, are not primarily designed as psychological interventions and may only partially address distress, maladaptive beliefs or coping difficulties. Our findings complement this literature by suggesting that formal psycho-oncology care, delivered by trained mental health professionals, may offer additional benefit for adherence, particularly in women with higher distress levels. The attenuation of survival hazard ratios after adjusting for adherence is compatible with the idea that improved medication taking may mediate part of any downstream benefit of psychological care.^[18]^ Given the observational nature of our data, this should be regarded as a hypothesis rather than proof of causality, but it provides a rationale for designing future trials that explicitly test adherence as a mediator.

From a service perspective, the results reinforce the view that psychological care should be integrated into routine breast cancer pathways rather than provided ad hoc or only on patient request.^[10,12]^ In settings with constrained resources, prioritizing early, structured interventions for patients with high or rising distress scores, complex psychosocial needs or early signs of adherence problems may be a realistic strategy. In addition, closer collaboration between oncology and psycho-oncology teams could facilitate timely identification of patients at risk and support the development of locally feasible care models, such as stepped-care or nurse-delivered interventions supervised by specialists.

Several limitations should be noted. First, as a retrospective study from a single tertiary center, the findings may not be generalizable to other institutions or healthcare systems, particularly those with different psycho-oncology resources or follow-up practices. We excluded patients who experienced events within 3 months after the surgery and adopted a 6-month milestone analysis. This reduced the time-related bias, but it might also result in insufficient representation of extremely high-risk patients, thereby overestimating the overall survival rate. Second, as a retrospective observational study, confounding by indication remains a central concern. While we employed rigorous methods including multivariable regression and IPTW to adjust for a wide range of measured demographic, clinical, and treatment-related covariates, these techniques can only account for observed confounders. They cannot adjust for unmeasured factors – such as patients’ social support networks, health literacy, financial status, personality traits, or intrinsic motivation to engage with healthcare – which may have influenced both the likelihood of receiving early structured psychological care and subsequent adherence to AET. This potential for residual confounding means that our findings represent strong associations, not proven causal effects.^[19,20]^

Third, distress was assessed using self-report instruments rather than structured clinical interviews, and information on psychotropic medication use was incomplete. Fourth, adherence was inferred from pharmacy dispensing data, which do not confirm actual ingestion, and our primary focus was on the first year of AET; longer-term persistence, dose changes and treatment switching were not examined in detail. Finally, survival analyses were based on relatively few events and a median follow-up of just over 2 years. These analyses should therefore be interpreted as exploratory and hypothesis-generating rather than definitive assessments of the effect of psychological care on OS and DFS.^[16,21]^ Given the limited number of survival events, the Cox regression and IPTW models may have the risk of overfitting, and the relevant results should be interpreted with caution.

## 5. Conclusions

In summary, among women undergoing surgery for stage I–III, HR+ breast cancer at a tertiary center, early structured postoperative psychological intervention was associated with better adherence to AET during the first year of treatment, particularly in patients with elevated distress. Any apparent survival advantage appeared modest and was estimated with considerable uncertainty. The data support further efforts to embed structured psycho-oncology services within routine postoperative follow-up as a means of addressing psychological needs and supporting sustained endocrine therapy use. Larger, prospective studies – ideally with randomized or quasi-experimental designs and longer follow-up – are needed to confirm these associations, clarify underlying mechanisms and define efficient, scalable models for integrating psychological care into breast cancer survivorship programs.

## Author contributions

**Conceptualization:** Rui Dong, Jingjing Yu.

**Data curation:** Rui Dong, Jingjing Yu.

**Formal analysis:** Rui Dong, Jingjing Yu.

**Funding acquisition:** Rui Dong, Jingjing Yu.

**Writing** – **original draft:** Rui Dong, Jingjing Yu.

**Writing** – **review & editing:** Rui Dong, Jingjing Yu.

**Investigation:** Jingjing Yu.
